# Urotropin, Methylene Citric Acid, and Urotropin Methylenecitrate (Helmitol or New-Urotropin)

**Published:** 1904-12

**Authors:** 


### ABSTRACT.

### Urotropin, Methylene Citric Acid, and Urotropin Methylenecitrate (Helmitol or New-urotropin).

Prof. Arthur Nicolaier (*Deutsches Archiv f.klin. Medicin,* Vol. 81, 1904,) records his very exhaustive studies of these drugs. It is an established fact that urotropin, introduced by him nine years ago, is a most efficient remedy, the number of original papers thereon exceeding 200. This literature confirms all the assertions he made in 1895,—that urotropin in 7½-grain doses dissolved in one half pint of water, given twice or thrice daily, is harmless and therapeutically effective; that soon after its administration it appears unchanged in the urine; that the urine, without losing its acidity, acquires uric acid solvent properties and an inhibitory action on microörganic growth; and, further, that there is often a diuresis.

It is used with good effect in uric acid concretions and in gout. But the most brilliant results are seen in bacterial diseases of the urinary passages, in cystitis, pyelitis, pyelonephritis, and in posterior urethritis. It lessons the pain and dysuria, eliminates the pus from the excretion and, if it is ammoniacal, removes the alkalinity and restores its acid reaction. It is ineffective only in tuberculosis of the urinary tract; and even here it is occasionally useful to combat mixed infection. Urotropin is generally effective in a short time; not infrequently all symptoms disappear permanently or do so when the remedy is resumed and persisted in for prolonged periods. Its efficacy is not dependent on a special urinary reaction; it acts equally well whether the urine is acid, alkaline or neutral. The very first case which I recorded was one of a very severe cystitis with ammoniacal urine.

Urotropin is indicated in all urinary infections, in bacteriuria, especially that of typhoid fever, which occurs in about 25 per cent. of all typhoid cases, usually after the third or fourth week. Urotropin, 7½ grains t. i. d., generally removes it in a week. As a prophylactic before and after instrumentation large daily doses up to 60 grains, should be given. Of course this is too much for steady use. Lately it has been recommended as a preventive of nephritis in scarlatina and, finally, it is successful in certain cases of phosphaturia.

Occasionally there occurs a bacterial affection of the urinary tract of nontubercular nature in which we do not get the desired effect from urotropin. This is in no way due to the reaction of the urine; the recalcitrant cases may be acid, neutral or alkaline.

Attempts have been made to find a preparation which is effectual even in these cases. While the sedative effect of the drug on the inflamed mucose is undoubtedly an important factor, its therapeutic action is chiefly dependent on the formaldehyde separation that occurs, and hence the endeavor has been to find a combination from which even greater amounts of formaldehyde would be separated.

In 1901 I experimented with the methylenecitrate of urotropin, which has been introduced under the names of helmitol and new urotropin. My first tests were made with methylene citric acid alone. Animal experiments showed that after its ingestion no free or loosely combined formaldehyde is present in the urine, but that it did contain some albumin. In a healthy man one gram four times daily occasioned severe diarrhea, and in another four grams caused a papulovesicular eczema of the skin and cheek. Even with the latter dose, urines infested with microörganisms and kept at a temperature of 98.6° F. became turbid in two days from abundant bacterial growth. Methylene citric acid, therefore, is decidedly inferior to urotropin. The same objection holds good for the sodium salt of methylene citric acid, citarin.

As to the methylene citric acid salt of urotropin (helmitol), which contains 40.7 per cent. urotropin, the usual daily dose is 45 to 60 grains, corresponding in urotropin content to 22½ grains urotropin. Larger doses of helmitol give unpleasant by-effects, such as meteorism and diarrhea, as Heuss, Mueller, Seifert, Fisch, and von Zellenburg have reported; these are doubtless due to the contained methylene citric acid. Urotropin in doses of 7½ grains t. i. d., dissolved in a tumblerful of water, never causes such untoward actions. The use of large doses of helmitol for prolonged periods is impossible, because of dysuria, vesical burning and irritation. Bering has observed renal irritation (albuminuria); Goldberg states that, in contradistinction to urotropin, 45 to 60 grains daily caused hematuria in two out of ten cases.

Like urotropin, helmitol does not affect the acidity of the urine. Both drugs simply hinder the growth of the microörganisms of ammoniacal fermentation, and hence the normal acid reaction reappears. After the exhibition of sufficiently large doses of helmitol, free or loosely combined formaldehyde is present in the urine. Since this is not the case after the ingestion of methylene citric acid alone, the question is whether this effect is not due to the 40.7 per cent. urotropin which helmitol contains.

Mueller made some comparative tests of urotropin and helmitol, from which he concludes that the latter has a greater antibacterial power; but he made the fundamental error of sterlising his urotropin and helmitol solutions by heat ere he added them to the nutrient media. I have shown that urotropin solutions split off formaldehyde when exposed to a high temperature; and this is far more so the case with helmitol solutions of equal strengths; for aqueous solutions of it react strongly acid, while those of urotropin are feebly alkaline. The helmitol solutions which Mueller added to the nutrient media contained 34 times as much formaldehyde as did the urotropin solutions; and this vitiates his experiments. Bruck made very extensive comparative investigations of the two drugs and concludes "that urotropin methylenecitrate (helmitol) is no more powerful than urotropin; its effect is dependent on the variety of bacteria and the amount of bacteria in the urine in exactly the same way; and that in very purulent urine it is decidedly weak." Clinical trials prove that in doses which are the coefficient of the usual urotropin doses, i. e., which are 2½ times as great as the latter, helmitol is effective in exactly the same diseases in which urotropin is effective and is ineffective in exactly the same cases in which urotropin is ineffective.

To conclude: (1) both in bacteriological and in animal experiment as well as in clinical tests methylene citric acid proved to be absolutely inert and to cause certain untoward action; (2) urotropin methylene citrate (helmitol) has been shown, both bacteriologically and clinically, to be dependent for its therapeutic efficacy upon its urotropin contents; (3) by reason of the contained methylene citric acid, helmitol may cause unpleasant by-effects; (4) as the dose of helmitol is based on its urotropin contents of 40.7 per cent., the dosage is more than twice as great and consequently more than doubly as expensive.

